# Operational strategies to deal with the COVID-19 emergency: recommendations from the Italian national society SIAGASCOT following the introduction of vaccines against the SARS-CoV-2 infection

**DOI:** 10.1007/s12306-023-00796-9

**Published:** 2023-09-02

**Authors:** Riccardo Compagnoni, Davide Cucchi, Raymond Klumpp, Mario Ronga, Massimo Berruto, Giovanni Di Giacomo, Pietro S. Randelli, SIAGASCOT “Guidelines” Work-group, Alessandro Carrozzo, Chiara Formigoni, Francesco Rosa, Fabio Sciancalepore

**Affiliations:** 1 1° Clinica Ortopedica, ASST Centro Specialistico Ortopedico Traumatologico Gaetano Pini-CTO, Piazza Cardinal Ferrari 1, 20122 Milan, Italy; 2 Department of Biomedical, Surgical and Dental Sciences, Università degli Studi di Milano, Via della Commenda 10, 20122 Milan, Italy; 3 Department of Orthopaedics and Trauma Surgery, Universitätsklinikum Bonn, Venusberg-Campus 1, 53127 Bonn, Germany; 4 Reparto di Ortopedia e Traumatologia dell`Ospedale di Treviglio-Caravaggio, ASST Bergamo Ovest, Piazzale Ospedale Luigi Meneguzzo, 24047 Treviglio, Italy; 5 Orthopaedic and Trauma Operative Unit, Department of Health Sciences, University of Eastern Piedmont, Novara - Italy. “Maggiore della Carità” Hospital, Corso Mazzini n.18, 28100 Novara, Italy; 6 Department of Orthopaedic and Trauma Surgery, Concordia Hospital for Special Surgery, 00145 Rome, Italy; 7 Laboratory of Applied Biomechanics, Department of Biomedical Sciences for Health, Università degli Studi di Milano, Via Mangiagalli 31, 20133 Milan, Italy

**Keywords:** Coronavirus, Vaccine, Orthopaedic, Pathways, SARS-CoV-2, Traumatology, Priorities, Strategies, Management

## Abstract

**Background:**

This article aims to present the operational recommendations adopted by the Italian national society for orthopaedic surgery, arthroscopy, and sports medicine (SIAGASCOT) in managing patients eligible to undergo elective orthopaedic surgery during the COVID-19 pandemic after the beginning of a national vaccination campaign.

**Materials and methods:**

An extensive literature search, analysing medical databases and scientific societies protocols, was performed to support this document. A four-step approach was used: 1—definition of priorities; 2—definition of significant clusters of interventions; 3—extraction of recommendations from international literature; and 4—adaptation of the recommendations to the specific features of the Italian healthcare system.

**Results:**

Three operational priorities were defined (“continuity of care and containment of the virus spread”, “examination of waiting lists”, and “definition of the role of vaccines”), six significant clusters of intervention were identified, and recommendations regarding the risk management for healthcare staff and hospital facility as well as the preoperative, in-hospital, and postoperative management were produced. Patient selection, preoperative screening, and pre-hospitalization procedures, which are regarded as pivotal roles in the safe management of patients eligible to undergo elective orthopaedic surgery, were analysed extensively.

**Conclusions:**

This document presents national-wide recommendations for managing patients eligible to undergo elective orthopaedic surgery with the beginning of the vaccination campaign. This paper could be the basis for similar documents adapted to the local healthcare systems in other countries.

**Level of evidence:**

Level IV.

**Supplementary Information:**

The online version contains supplementary material available at 10.1007/s12306-023-00796-9.

## Bullet points

What is already known:

- The COVID-19 pandemic had dramatic consequences on prevention, diagnosis, and treatment of musculoskeletal pathologies.
- Improvements on virus knowledge led to enhanced treatment and development of safe and effective vaccines.

What are the new findings:

- Continuity of care and containment of the virus spread and examination of waiting lists are priorities after the beginning of a national vaccination campaign.
- Patient selection, preoperative screening, and pre-hospitalization procedures should be well organized to allow effective treatment and resource allocation.

## Background

Since the first report of pneumonia cases of unknown aetiology detected in Wuhan City, Hubei, China, at the end of 2019 [28], several countries have experienced pneumonia outbreaks due to a novel and highly contagious coronavirus, later named SARS-CoV-2 [30]. In Italy, the state of emergency to limit diffusion of the virus was declared on the 31st of January 2020 [5] with dramatic consequences on the prevention, diagnosis, and treatment of musculoskeletal pathologies. Orthopaedic and trauma surgeries were replanned to ensure emergency surgical treatment for non-deferrable trauma cases and continuity of care for not postponable musculoskeletal pathology (Table 1).

**Table 1:** Non-postponable musculoskeletal pathology of malignant tumours

Peri-articular tumours at risk of fracture
Musculoskeletal pathology resulting in neurological deficits
Avascular necrosis and destructive arthropathies
Acute traumatic tendon injuries
Prosthetic dislocation
Severe loosening of prosthetic implants
Loose bodies causing acute joint locking

To provide national-wide recommendations for the management of patients eligible to undergo elective orthopaedic surgery in this unexpected and exceptional health situation, operational strategies were published by the national society for orthopaedic surgery, arthroscopy and sports medicine SIAGASCOT (Società Italiana Artroscopia—Ginocchio—Arto superiore—Sport—Cartilagine—Tecnologie Ortopediche) in May 2020 [9]. This document was intended to give support to clinicians in selecting and managing patients eligible for elective orthopaedic surgery while safeguarding both their health and those of the healthcare professionals. Three years have now passed since the beginning of the pandemic. Dramatic improvements on the knowledge of virus biology have occurred, leading to enhanced patient treatment and the development of safe and effective vaccines [23]: therefore, considering these scientific achievements, the evolution of diagnostic-therapeutic strategies and the beginning of the vaccination campaign, an update of the initially proposed recommendations has been diffused in the Italian language to all clinicians affiliated to the SIAGASCOT and is available online [9]. This article aims to present, describe and explain these new operational strategies, offering international readers the possibility to benefit from several lessons learned during the Italian COVID-19 experience.

## Materials and methods

The definition of different phases of the COVID-19 pandemic was based on that used in the Italian presidential and ministerial decrees, which follows the timeline of the disease. Phase 1 was defined as the phase in which the virus spread was rapid and out of control, with severe effects on the health system; Phase 2 is characterized by stability of infections and the absence of extreme overload of health facilities. Phase 3 is defined by the arrival of a cure that will efficiently eradicate the disease and permit the return to everyday life, and Phase 4 is when the pandemic is over [17]. With the beginning of the vaccination campaign, Europe can be considered in transition between phase 2 and phase 3.

After extraction of relevant literature evidence (Online Appendix A), a four-step approach was used to create and update the recommendations [8, 16, 19, 24, 27, 29]:

1. Definition of priorities, depending on the specific phase of the COVID-19 pandemic.
2. Definition of major clusters of interventions and grouping of relevant literature to each cluster.
3. Extraction of recommendations from international literature.
4. Adaptation of the recommendations to the specific features of the Italian healthcare system and to the legal obligations and requirements of each specific phase of the COVID-19 pandemic.

The development of this recommendations was endorsed by the society SIAGASCOT.

## Results

### Operational priorities after the beginning of the COVID-19 vaccination campaign

#### Continuity of care and containment of the virus spread

One of the most relevant aspects of healthcare planning in this phase is expanding the diagnostic, inpatient, and outpatient treatment capacity for diseases not directly related to the SARS-CoV-2 infection, to ensure treatment continuity and avoid later sequelae of these diseases. This aspect was sacrificed at the beginning of the COVID-19 pandemic to reduce perioperative and in-hospital infections related to a more than tenfold increase in mortality [10] and direct resources to treat infected patients. With the beginning of the vaccination campaign, it appears essential to re-establish this critical pillar of the national health system.

#### Examination of waiting lists

The conversion of many hospitals to predominantly treat COVID-19 patients has caused a drastic downsizing of elective orthopaedic activity during the last year and led to a progressive lengthening of waiting lists. This has two significant consequences: first, there is an increase in the overall number of patients requiring surgical treatment and, therefore, candidates for hospitalization for elective orthopaedic surgery. Second, there is a lengthening of the interval between the time of the surgical indication and the day of actual hospitalization. This gives rise to both organizational and clinical problems. The first is related to the definition of treatment priorities and the need for reprogramming procedures; the latter is related to the need to re-evaluate patients suffering from potentially evolutive pathologies, which could have considerably evolved over the long waiting period.

#### Definition of the role of vaccines

The recent use of vaccines to eradicate the pandemic has raised hopes in the scientific community and the general population. Up to the 27th of March 2023 in the European Community, seven vaccines are authorized by the EMA (European Medicines Agency) [7], and more than 140 million doses have been administered in Italy and more than 975 Million in the European community [12].

Regarding orthopaedic surgery, vaccines could facilitate several aspects of managing patients eligible for elective surgery in the near future, raising therefore great expectations in the orthopaedic community.

### Major clusters of intervention

The following six major clusters of intervention were identified, based on the priorities set in other countries and previous epidemics and pandemics [8, 16, 19, 24, 27, 29]:

1. A—Risk management for healthcare staff and hospital facility
2. B—Preoperative management: patient selection
3. C—Preoperative management: preoperative screening
4. D—Preoperative management: pre-hospitalization procedures
5. E—In-hospital management
6. F—Postoperative management

These recommendations are illustrated in the following six sections named after each major intervention cluster.

## Section A

### Risk management for staff and hospital facility

#### General recommendations

The local epidemiology of COVID-19 must be considered before resuming elective orthopaedic surgery to ensure a safe path of care for patients and healthcare professionals. Furthermore, specific characteristics of each healthcare facility must be considered carefully, in terms of both structural resources and personnel. It is also recommended to identify decision-making roles and establish priority management protocols before starting the surgical activity. In case of an increase in the number of infections, the possibility of a timely reduction up to complete stop of all elective surgeries must be considered.

#### Characteristics of the structures in which to perform elective orthopaedic surgery

Elective orthopaedic activity should be resumed in facilities dedicated exclusively to non-COVID patients or that can at least guarantee physically distinct paths and spaces between COVID-positive and COVID-negative patients [16]. These separate paths should be guaranteed from the pre-hospitalization phase to discharge, also considering the rehabilitation process following hospital discharge. A path that ensures the physical separation of patients negative for SARS-CoV-2 diagnostic tests for the entire duration of treatment can be defined as COVID-FREE.

#### Basic requirements for healthcare professionals

The correct use of personal protective equipment (PPE) and the compliance with hygienic rules during the stay of the staff inside the facility minimize the risk of virus transmission. Healthcare personnel must keep a surgical mask for the entire stay within the facility, even if not directly involved in patient care. Specific precautions must be taken in the operating room and during the perioperative process depending on the performed procedure [24]. COVID-19 vaccination of health personnel is recommended. Nevertheless, having received vaccination against the SARS-CoV-2 infection does not exempt hospital staff from compliance with hygiene regulations and correct use of PPE.

#### PPE for patients

In addition to the recommendations for healthcare professionals, patients must always wear a surgical mask throughout their hospital stay. Having received a vaccination against the SARS-CoV-2 infection does not exempt from compliance with hygiene regulations and the correct use of PPE.

#### SARS-CoV-2 testing for healthcare professionals

Depending on local legislation and the availability of diagnostic tests, it is encouraged to consider testing health personnel periodically using the COVID-RT-PCR test to limit in-hospital transmission [2] and decide on the possible creation of medical and nursing teams dedicated to the management of non-infected patients only.

#### Risk of disease transmission during surgical procedures

The viral concentration of SARS-CoV-2 in bones, joints, periarticular tissues, and body fluids of infected patients is currently unknown. However, it is reasonable to assume that it is lower in musculoskeletal tissues than in respiratory or digestive tissues. Given these uncertainties, care is recommended in procedures that generate high aerosol production through electrocautery devices, oscillating saw, and pulsed lavage.

#### Patient awareness

The patient's behaviour during the perioperative procedures and the hospital stay is of utmost importance to limit the spread of the infection, protect healthcare professionals and reduce the resource commitment of the whole health system. Therefore, healthcare personnel must emphasize the need to comply with the national and local regulations on social distancing or isolation and correct use of PPE at every stage of the diagnostic and therapeutic process.

## Section B

### Preoperative management: patient selection

#### Identification and selection of patients

Preoperative management plays a fundamental role in guaranteeing the safety of elective orthopaedic surgery both for patients and healthcare professionals. The primary purpose of preoperative management is to ensure with reasonable safety that no in-hospital infection occurs. This can be obtained by reducing the possibility that positive, however asymptomatic patients enter a COVID-FREE path.

Minimizing the patient's hospitalization time is an effective way to achieve this goal and optimize hospital resources, which becomes possible with careful patient selection and preparation. A multidisciplinary assessment with the Department of Anaesthesiology/Intensive Care Medicine (responsible for the final authorization to the surgical procedure) is fundamental to select patients in the preoperative phase appropriately.

#### General principles

Due to comorbidities and type of intervention, all patients who need to be hospitalized in a medium–/high-intensity care setting in the postoperative period should be excluded from elective orthopaedic surgery during this phase and should therefore be postponed. Advanced biological age, obesity, and disabling chronic cardiovascular and respiratory diseases are, for example, some relevant intrinsic risk factors for the need for medium–/high-intensity postoperative care. When considering surgical variables, more invasive procedures of prolonged duration, procedures with an increased risk of blood transfusion and procedures where anaesthesiologic procedures and especially locoregional analgesia are more difficult should be considered at greater risk of hospitalization in a medium–/high-intensity care setting in the postoperative period.

#### Anaesthesiologic considerations

Priority should be given to patients of ASA I and ASA II classes for whom a prolonged postoperative course of postoperative hospitalization in a medium–/high-intensity setting is less likely. ASA III patients may be considered if they need to undergo a procedure for which medium–/high-intensity postoperative monitoring is not expected.

For example, knee prostheses' revision can be considered a highly complex intervention with a greater risk of periprocedural problems, which should be reserved for a carefully selected and healthy population (ASA I and II). On the contrary, low complexity and minimally invasive intervention (for example, arthroscopic meniscectomy) can also be proposed in patients with more disabling comorbidities (ASA III) for whom, thanks to different anaesthesiologic approaches such as techniques of locoregional anaesthesia (spinal anaesthesia or peripheral block), adequate results can be achieved safely without necessarily requiring postoperative monitoring in a medium–/high-intensity care setting.

#### Logistic and social considerations

It is advisable to avoid carrying out elective orthopaedic surgical interventions on patients who, due to clinical, cognitive, or social characteristics, require the constant presence of a caregiver or special assistance during hospitalization.

#### Recommendations on the typology of surgical interventions

As far as is known, by combining the criteria of young age, absence of comorbidities and minimally invasive or arthroscopic surgery, it is possible to select low-risk patients and procedures.

To limit the risk of infection spread and the occupation of hospital beds, surgical procedures that require a hospital stay of no more than 3 days are recommended. Therefore, it is recommended that the surgical team evaluates which procedures require a hospital stay of 3 days or less in consultation with the team of anaesthesiologists/pain therapists and rehabilitators.

#### Management of the waiting lists

The increase in the number of patients who require surgical treatment and the lengthening of the time interval between indication for surgery and hospitalization determine both an organizational and a clinical problem. The first is related to the definition of treatment priorities and the need for reprogramming procedures; the latter is related to the need to re-evaluate patients suffering from potentially evolutive pathologies, which may have considerably evolved during the long waiting period.

A first aspect that must be considered is the patient's treatment priority level which may have changed category over time (e.g. functional reduction and pain worsening). Clinical re-evaluation of the patient, integrated by new radiological/instrumental exams if deemed necessary, is therefore recommended in addition to the use of validated assessment scales for pain and function (for example, the numerical rating scale, NRS), which can be administrated during a telephone triage by adequately trained nursing staff.

The clinical re-evaluation of the patients that are on a waiting list for a long time, which must be carried out in compliance with the aforementioned hygienic rules, is necessary to confirm or not the surgical indication defined previously. The indication may have changed due to the evolution of the disease (e.g. rotator cuff injury, which has become irreparable) or to an increased intraoperative risk caused by changes in the patient's health status.

## Section C

### Preoperative management: patient screening

#### Type of patients

Shortly after the beginning of the COVID-19 pandemic, Fineberg proposed a classification of patients in 5 categories based on exposure to SARS-CoV-2 [15], which the ESSKA implemented with a sixth category which also considered comorbidities [24]. The significant number of patients who have been infected by COVID-19 with different symptoms within the last year, with or without being tested, and the increasingly extensive vaccination campaign leads to further heterogeneity among patients eligible for elective orthopaedic surgery, requiring a new approach to stratify the risk. In order to simplify and effectively allocate resources for evaluation of perioperative risk resulting from COVID-19 sequelae, a more straightforward categorization in two broader groups is now proposed:

#### Patients who have not developed pathognomonic symptoms of COVID-19

This group comprises two sub-categories: patients who did not have contact with the virus and infected patients who did not show symptoms.

All patients undergoing elective orthopaedic surgery must undergo a COVID-RT-PCR test 48–72 h prior to surgery during the pre-hospitalization. Social isolation at home is recommended in the time frame between the execution of the test and hospitalization.

An immunoassay/serology test can be performed if permitted and available before surgery to improve preoperative risk assessment, considering that at least 17% of the patients contacting COVID-19 are asymptomatic [4].

In case of a positive COVID-RT-PCR test, any elective surgery should be deferred until proven healing, as defined according to current clinical and laboratory criteria.

In case of a positive immunoassay/serology test and negative COVID-RT-PCR test, patients may undergo elective orthopaedic surgery but should follow the protocol described for group two.

#### Patients who have developed the COVID-19 disease

This heterogeneous group includes patients who have developed the disease confirmed by laboratory tests and/or clinical expressions of different magnitude and severity. Current scientific evidence does not clearly define the degree of risk in patients who recovered from infection and candidates for elective orthopaedic surgery, but a fundamental aspect is to evaluate possible organ damage resulting from SARS-CoV-2 [6, 21].

Prior to any elective orthopaedic surgery, all patients belonging to Group 2 should undergo two COVID-RT-PCR, both with negative results, the latter of which obtained not earlier than 72 h prior to surgery during the pre-hospitalization.

Social isolation at home is recommended in the time frame between the execution of the test and hospitalization.

Furthermore, these patients should undergo an immunoassay/serology test if permitted and available before surgery and possibly lung imaging according to local protocols before elective surgery. A multidisciplinary assessment for the staging of the perioperative risk resulting from COVID-19 sequelae is recommended for these patients, especially if comorbidities were present before infection.

In case of a positive COVID-RT-PCR test, any elective surgery should be deferred until proven healing, as defined according to current clinical and laboratory criteria.

Current scientific data are insufficient to accurately define the duration of immunity acquired by patients who recovered from COVID-19. The appearance of new variants of the virus and of cases of re-infections in healed and vaccinated patients make the interpretation of the data particularly complex. In this regard, a recently published study showed that protection against repeated infection was given in 80.5% of the whole study population, but only 47.1% among those aged 65 years or older [18]. Regarding the vaccinated population, disease protection of 63% in partial vaccination cycle [3] and about 90% in case of complete vaccination cycle [11] has been reported. However, the duration of immunity acquired and the incidence of virus carriers in these subjects remain still unclear.

## Section D

### Preoperative management: pre-hospitalization procedures

Pre-hospitalization procedures are fundamental to avoid the undesired hospitalization of COVID-positive patients within a COVID-FREE path. A preoperative questionnaire, which must include an evaluation of all the signs and symptoms associated with COVID-19 (fever, cough, other respiratory symptoms, alteration of the senses of taste and smell, gastrointestinal symptoms, skin rash, and muscle soreness), must be administered before the beginning of the pre-hospitalization procedures by telephone or by other contactless ways (Online Appendix B).

Considering the problems related to the management of the waiting lists described above, it is recommended to integrate the pre-hospitalization exams with a careful clinical re-evaluation, completed by new radiological/instrumental exams if deemed necessary. The use of technological means that do not require the presence of the patient at the healthcare institution can be considered; nevertheless, a clinical re-evaluation of patients who are on a waiting list for a long time is recommended to confirm the surgical indication defined previously. Other diagnostic tests (for example, chest X-ray, or pulmonary CT) can be performed at this stage, based on the outcome of the questionnaire, previous SARS-CoV-2 exposure, available SARS-CoV-2 test results and a detailed patient interview.

During pre-hospitalization, COVID-19-RT-PCR and/or immunological/serological tests may be performed according to the patient and local protocols. This assessment must take place 48–72 h before hospitalization. All candidates for elective surgery must undergo COVID-19-RT-PCR screening no earlier than 72 h before surgery. In patients already declared healed from COVID-19 disease or for whom 90 days have passed from the onset of the disease or the first positive test, the positivity of the COVID-19-RT-PCR screening should be considered as possible reinfection, and the subject should be consequently put in isolation and the intervention suspended until negativization.

At the end of the pre-hospitalization, the patient may be discharged at home or hospitalized. In case of discharge at home, it is helpful to recommend social isolation within the time frame between executing the COVID-19-RT-PCR test during pre-hospitalization and hospitalization. Admission to the hospital directly after performing pre-hospitalization and COVID-19-RT-PCR test is possible only if isolation of each patient in a single room is guaranteed until the results of the COVID-19-RT-PCR test are available. It is advisable to evaluate the management of pre-hospitalization on an outpatient basis.

Body temperature must be monitored until the day of surgery and at the time of admission, using a specific checklist (Online Appendix B).

In case of temporary ineligibility for surgery for reasons other than exposure to SARS-CoV-2, it is necessary to repeat the entire pre-hospitalization, including the COVID-19-RT-PCR tests and possibly the diagnostic imaging tests deemed necessary.

## Section E

### In-hospital management

The available evidence suggests that patients in a hospital setting, regardless of their risk group, should be managed following COVID-FREE pathways [16].

Upon admission, the patient must be re-evaluated using a dedicated checklist to identify the possible appearance of signs and/or symptoms of SARS-CoV-2 infection (Online Appendix B).

Despite periodic fluctuations, the prevalence of COVID-19 remains high in Europe. Therefore, the onset of postoperative fever or the appearance of one of the symptoms indicated in the previously proposed checklist (Online Appendix B) must activate a path of differential diagnostics that takes into consideration the possibility of in-hospital SARS-CoV-2 infection, with consequent patient isolation and specialist consultation, COVID-19-RT-PCR tests, and appropriate diagnostic imaging according to regional recommendations and local protocols. This also holds true in patients who have previously developed COVID-19 disease and were considered as healed and in patients who have received vaccines against the SARS-CoV-2 infection.

Epidemiological data and those derived from post-COVID-19-disease and post-vaccine serological surveillance will allow us to better understand the protective effect of the generated antibodies. Until then, scrupulous observance of the protection and prevention measures that have been put in place since the beginning of the pandemic remains a must, even for vaccinated subjects, including the correct use of PPE.

It is advisable to adopt an additional informed consent form for hospital admission that includes the description and possible consequences of the SARS-CoV-2 infection, which should be adapted to the local situation and the procedures of the individual healthcare facility.

## Section F

### Postoperative management

Postoperative management must be discussed before surgery by a multidisciplinary team composed of the surgical, anaesthesiologic, and rehabilitative teams and standardized as much as possible. The social and family context of the patient must be taken into consideration, giving priority to access elective orthopaedic surgery to those patients who can carry out the rehabilitation entirely at home in a self-assisted way or with the support of appropriate physical and/or multimedia material.

It is recommended to avoid carrying out elective orthopaedic surgical interventions on patients who require constant assistance during post-surgical rehabilitation procedures due to their clinical, cognitive, or social characteristics,

Postoperative follow-up should be carried out, if possible, with the use of telemedicine to minimize postoperative visits and thus limiting patient movement. Outpatient follow-up visits (e.g. wound dressings, removal of sutures) must be agreed on if possible before surgery and at the latest at the time of discharge. Whenever variations in the surgical technique make it possible to reduce the frequency of outpatient controls without creating additional risks for the patient (e.g. using resorbable skin sutures or advanced wound dressings), they are advisable and should be considered by the surgical team.

With an accurate description of the postoperative pathway, complete planning must be made available and precisely discussed with the patient before any surgery. Postoperative appointments must include detection of potential COVID-19-related complications. To protect healthcare personnel and patients, the correct use of PPE and measures to reduce the risk of contagion by an asymptomatic positive patient is essential during outpatient follow-up visits. The appointment agendas and the organization of the clinic must be structured in such a way as to ensure spatial and temporal distancing between patients.

In case of discharge of the patient to a rehabilitation clinic, a COVID-19-RT-PCR test should be performed before transferring the patient, to minimise the risk of viral spread in rehabilitation clinics.

In the approach to postoperative management, it is helpful to remember that the patient's behaviour is of utmost importance to limit the spread of the disease, protect healthcare professionals and reduce the resource commitment of the whole health system. Therefore, healthcare personnel must emphasize, even at this stage, the need to comply with the national and local regulations on social distancing or isolation and correctly use PPE.

## Discussion

The COVID-19 pandemic has had a profound impact on the world, touching almost every aspect of human life and most significantly impacting global health—orthopaedic surgery was also greatly affected, in all its subspecialities [1, 13, 14, 22]. The development of vaccines could reduce the enormous strain on healthcare systems and healthcare workers and helped countries recover from the economic consequences of the pandemic. To optimize the management of patients eligible to undergo elective orthopaedic surgery in this unexpected and exceptional health situation, several national-wide or international recommendations for operational strategies were published [20, 24–26]. Among these, SIAGASCOT presented the first Italian operational guidelines in May 2020, subsequently updated in April 2021 [9]. This document was intended to give support to clinicians in selecting and managing patients eligible for elective orthopaedic surgery while safeguarding both their health and those of the healthcare professionals. This document dealt also with the evolution of diagnostic-therapeutic strategies and the beginning of the vaccination campaign being as such a basis for development of guidelines in other countries or for future pandemics [9].

## Conclusion

This document presents national-wide recommendations for managing patients eligible to undergo elective orthopaedic surgery with the beginning of the vaccination campaign. The clinical implication of this document is the potential reduction of SARS-CoV-2 infections within patients eligible to undergo elective orthopaedic surgery and the optimisation of their surgical and postoperative treatment. Furthermore, it represents an important update for Italian orthopaedics, offering national recommendations as available in other countries, but specifically adapted taking into account the national reality and its own peculiarities. Furthermore, this paper could be the basis for similar documents adapted to the local healthcare systems in other countries. Since the knowledge on SARS-CoV-2 pathology is currently limited, fragmentary, and in continuous evolution, the validity of the protocols proposed in this phase is to be considered limited in time.

### Supplementary Information

Below is the link to the electronic supplementary material.

Supplementary file1 (DOC 36 kb)

Supplementary file2 (DOC 38 kb)
