# Supplementary material for: Operational strategies to deal with the COVID-19 emergency: recommendations from the Italian national society SIAGASCOT following the introduction of vaccines against the SARS-CoV-2 infection
Source: Musculoskelet Surg. 2023 Sep 2;107(4):471–9. doi: 10.1007/s12306-023-00796-9 (PMC10709259; doi:10.1007/s12306-023-00796-9)
Supplement: Supplementary file 1 — Supplementary file1 (DOC 36 kb) [file 12306_2023_796_MOESM1_ESM.doc]

Appendix A

***Review search strategy***

MEDLINE/PUBMED

| Search | Query | Items |
| --- | --- | --- |
| #7 | Limit Publication date from 2020/01/01 | 125 |
| #6 | Search (((((((("COVID-19 vaccine"[Supplementary Concept] OR "COVID-19 diagnostic testing"[Supplementary Concept] OR "COVID-19 serotherapy"[Supplementary Concept] OR "spike glycoprotein, COVID-19 virus"[Supplementary Concept] OR "COVID-19"[Supplementary Concept] OR "severe acute respiratory syndrome coronavirus 2"[Supplementary Concept] OR "COVID-19 drug treatment"[Supplementary Concept])) OR "Coronavirus Infections"[Mesh])) OR ((((("Wuhan seafood market pneumonia virus"[Text Word] OR COVID*[Text Word] OR "COVID19"[Text Word] OR "COVID 19"[Text Word] OR "COVID-19"[Text Word] OR "coronavirus disease 2019"[Text Word] OR "SARS-CoV-2"[Text Word] OR "SARS-CoV2"[Text Word] OR SARS2[Text Word] OR "2019-nCoV"[Text Word] OR "2019 novel coronavirus"[Text Word] OR "severe acute respiratory syndrome coronavirus 2"[Text Word] OR "2019 novel coronavirus infection"[Text Word] OR "coronavirus disease 2019"[Text Word] OR "coronavirus disease-19"[Text Word] OR "novel coronavirus"[Text Word] OR coronavirus*[Text Word] OR "SARS-CoV-19"[Text Word] OR "SARS-CoV-2019"[Text Word] OR betacoronavirus*[Text Word] OR beta-coronavirus*[Text Word]))))))))) AND ((((("Orthopedics"[Mesh] OR "Orthopedic Procedures"[Mesh] OR "Orthopedic Surgeons"[Mesh] OR "Elective Surgical Procedures"[Mesh] OR "Anesthetists"[Mesh] OR "Anesthesiologists"[Mesh] OR "Anesthesia"[Mesh]))) OR "Bone Neoplasms"[Mesh]) OR ((((orthop*[Text Word] OR arthroplast*[Text Word] OR ostheo*[Text Word] OR traumatolog*[Text Word] OR fracture*[Text Word] OR "knee prosthesis"[Text Word] OR "hip prosthesis"[Text Word] OR orthoger*[Text Word] OR ortho-ger*[Text Word] OR "elective surgery"[Text Word] OR "elective surgeries"[Text Word] OR anesthes*[Text Word] OR elective-surg*[Text Word]))) OR (((hip*[Text Word] OR knee*[Text Word] OR femur*[Text Word] OR femoral*[Text Word] OR bone*[Text Word] OR ligament*[Text Word])) AND (fracture*[Text Word] OR prosthes*[Text Word] OR surg*[Text Word] OR cancer*[Text Word] OR tumor*[Text Word] OR tumour*[Text Word] OR carcinoma*[Text Word] OR sarcoma*[Text Word] OR neoplasm*[Text Word] OR oncol*[Text Word])))))) | 265 |
| #5 | Search (((("Orthopedics"[Mesh] OR "Orthopedic Procedures"[Mesh] OR "Orthopedic Surgeons"[Mesh] OR "Elective Surgical Procedures"[Mesh] OR "Anesthetists"[Mesh] OR "Anesthesiologists"[Mesh] OR "Anesthesia"[Mesh]))) OR "Bone Neoplasms"[Mesh]) OR ((((orthop*[Text Word] OR arthroplast*[Text Word] OR ostheo*[Text Word] OR traumatolog*[Text Word] OR fracture*[Text Word] OR "knee prosthesis"[Text Word] OR "hip prosthesis"[Text Word] OR orthoger*[Text Word] OR ortho-ger*[Text Word] OR "elective surgery"[Text Word] OR "elective surgeries"[Text Word] OR anesthes*[Text Word] OR elective-surg*[Text Word]))) OR (((hip*[Text Word] OR knee*[Text Word] OR femur*[Text Word] OR femoral*[Text Word] OR bone*[Text Word] OR ligament*[Text Word])) AND (fracture*[Text Word] OR prosthes*[Text Word] OR surg*[Text Word] OR cancer*[Text Word] OR tumor*[Text Word] OR tumour*[Text Word] OR carcinoma*[Text Word] OR sarcoma*[Text Word] OR neoplasm*[Text Word] OR oncol*[Text Word]))))) | 1316745 |
| #4 | Search (((orthop*[Text Word] OR arthroplast*[Text Word] OR ostheo*[Text Word] OR traumatolog*[Text Word] OR fracture*[Text Word] OR "knee prosthesis"[Text Word] OR "hip prosthesis"[Text Word] OR orthoger*[Text Word] OR ortho-ger*[Text Word] OR "elective surgery"[Text Word] OR "elective surgeries"[Text Word] OR anesthes*[Text Word] OR elective-surg*[Text Word]))) OR (((hip*[Text Word] OR knee*[Text Word] OR femur*[Text Word] OR femoral*[Text Word] OR bone*[Text Word] OR ligament*[Text Word])) AND (fracture*[Text Word] OR prosthes*[Text Word] OR surg*[Text Word] OR cancer*[Text Word] OR tumor*[Text Word] OR tumour*[Text Word] OR carcinoma*[Text Word] OR sarcoma*[Text Word] OR neoplasm*[Text Word] OR oncol*[Text Word])))) | 1189047 |
| #3 | Search "Bone Neoplasms"[Mesh] | 125259 |
| #2 | Search ("Orthopedics"[Mesh] OR "Orthopedic Procedures"[Mesh] OR "Orthopedic Surgeons"[Mesh] OR "Elective Surgical Procedures"[Mesh] OR "Anesthetists"[Mesh] OR "Anesthesiologists"[Mesh] OR "Anesthesia"[Mesh]) | 516512 |
| #1 | Search (((((("COVID-19 vaccine"[Supplementary Concept] OR "COVID-19 diagnostic testing"[Supplementary Concept] OR "COVID-19 serotherapy"[Supplementary Concept] OR "spike glycoprotein, COVID-19 virus"[Supplementary Concept] OR "COVID-19"[Supplementary Concept] OR "severe acute respiratory syndrome coronavirus 2"[Supplementary Concept] OR "COVID-19 drug treatment"[Supplementary Concept])) OR "Coronavirus Infections"[Mesh])) OR ((((("Wuhan seafood market pneumonia virus"[Text Word] OR COVID*[Text Word] OR "COVID19"[Text Word] OR "COVID 19"[Text Word] OR "COVID-19"[Text Word] OR "coronavirus disease 2019"[Text Word] OR "SARS-CoV-2"[Text Word] OR "SARS-CoV2"[Text Word] OR SARS2[Text Word] OR "2019-nCoV"[Text Word] OR "2019 novel coronavirus"[Text Word] OR "severe acute respiratory syndrome coronavirus 2"[Text Word] OR "2019 novel coronavirus infection"[Text Word] OR "coronavirus disease 2019"[Text Word] OR "coronavirus disease-19"[Text Word] OR "novel coronavirus"[Text Word] OR coronavirus*[Text Word] OR "SARS-CoV-19"[Text Word] OR "SARS-CoV-2019"[Text Word] OR betacoronavirus*[Text Word] OR beta-coronavirus*[Text Word]))))))) | 24825 |
