# Supplementary material for: Operational strategies to deal with the COVID-19 emergency: recommendations from the Italian national society SIAGASCOT following the introduction of vaccines against the SARS-CoV-2 infection
Source: Musculoskelet Surg. 2023 Sep 2;107(4):471–9. doi: 10.1007/s12306-023-00796-9 (PMC10709259; doi:10.1007/s12306-023-00796-9)
Supplement: Supplementary file 2 — Supplementary file2 (DOC 38 kb) [file 12306_2023_796_MOESM2_ESM.doc]

Appendix B

Anamnestic questionnaire, telephonic screening interview for COVID-19, hospitalization checklist

**Anagraphic and biometric data**

Name: ________________________ Surname: ________________________

Age: ____________ Gender: □ Female □ Male

Weight _____ Kg Height _____cm BMI ______ kg/m^2^

**Generic anamnestic questionnaire**

Allergy drugs/latex/other □ Yes □ No

Smoking habits □ Yes □ No

Neurological diseases □ Yes □ No

Cognitive impairment □ Yes □ No

Psychiatric disorders □ Yes □ No

Cardiovascular diseases □ Yes □ No

Respiratory diseases □ Yes □ No

Diabetes mellitus □ Yes □ No

Renal impairment □ Yes □ No

Liver disease □ Yes □ No

Active oncologic pathology □ Yes □ No

Other chronic disease □ Yes □ No

Annotations: _____________________________________________________________________

________________________________________________________________________________

**Specific anamnestic questionnaire for COVID-19**

Area of residence / domicile: ________________

1. Did you test positive for COVID-19 (SARS-CoV-2)? □ Yes □ No

2. Did anybody living at your home test positive for COVID? □ Yes □ No

3. Did you have contact with anybody that had COVID-19 infection (from

2 days before of symptoms appearance till 20 days after infection)? □ Yes □ No

4. Do you live in contact with anybody aged more than 65 years with

severe health issues or who has been considered at risk for COVID-19? □ Yes □ No

5. Did you pay visit to anybody in a nursing home during the last 30 days? □ Yes □ No

6. During the last 30 days, did you present one or more of the following

signs/symptoms mentioned in pre-hospitalization checklist? □ Yes □ No

PRE-HOSPITALIZATION CHECKLIST

Beginning of symptoms, date: ..…./……./…….

Fever >37,2° □ Yes □ No

Cough □ Yes □ No

Fatigue □ Yes □ No

Sore throat □ Yes □ No

Headache □ Yes □ No

Loss of taste □ Yes □ No

Loss of smell □ Yes □ No

Muscle soreness □ Yes □ No

Nasal congestion □ Yes □ No

Diarrhoea □ Yes □ No

Skin rash □ Yes □ No

*HOSPITALIZATION CHECKLIST*

*Fever >37,2° □ Yes □ No*

*Cough □ Yes □ No*

*Fatigue □ Yes □ No*

*Sore throat □ Yes □ No*

*Headache □ Yes □ No*

*Loss of taste □ Yes □ No*

*Loss of smell □ Yes □ No*

*Muscle soreness □ Yes □ No*

*Nasal congestion □ Yes □ No*

*Diarrhoea □ Yes □ No*

*Skin rash □ Yes □ No*

Temperature measurement at the moment of hospitalization:__________ ° C
